# Intertumoral heterogeneity in patient-specific drug sensitivities in treatment-naïve glioblastoma

**DOI:** 10.1186/s12885-019-5861-4

**Published:** 2019-06-25

**Authors:** Erlend Skaga, Evgeny Kulesskiy, Artem Fayzullin, Cecilie J. Sandberg, Swapnil Potdar, Aija Kyttälä, Iver A. Langmoen, Aki Laakso, Emília Gaál-Paavola, Markus Perola, Krister Wennerberg, Einar O. Vik-Mo

**Affiliations:** 1 Vilhelm Magnus Laboratory for Neurosurgical Research, Institute for Surgical Research and Department of Neurosurgery, Oslo University Hospital, P.O. Box 4950 Nydalen, 0424 Oslo, Norway; 2 Institute of Clinical Medicine, Faculty of Medicine, University of Oslo, P.O. Box 1112 Blindern, 0317 Oslo, Norway; 3 Institute for Molecular Medicine Finland, FIMM, University of Helsinki, Tukholmankatu 8, 00290 Helsinki, Finland; 4 National Institute for Health and Welfare, Genomics and Biomarkers Unit, P.O. Box 30, FI-00271 Helsinki, Finland; 5 Department of Neurosurgery, Helsinki University Hospital and Clinical Neurosciences, University of Helsinki, Topeliuksenkatu 5, 00260 Helsinki, Finland

**Keywords:** Glioblastoma, Glioblastoma stem cells, High-throughput drug screening, Individualized medicine, Drug sensitivity, Functional precision medicine

## Abstract

**Background:**

A major barrier to effective treatment of glioblastoma (GBM) is the large intertumoral heterogeneity at the genetic and cellular level. In early phase clinical trials, patient heterogeneity in response to therapy is commonly observed; however, how tumor heterogeneity is reflected in individual drug sensitivities in the treatment-naïve glioblastoma stem cells (GSC) is unclear.

**Methods:**

We cultured 12 patient-derived primary GBMs as tumorspheres and validated tumor stem cell properties by functional assays. Using automated high-throughput screening (HTS), we evaluated sensitivity to 461 anticancer drugs in a collection covering most FDA-approved anticancer drugs and investigational compounds with a broad range of molecular targets. Statistical analyses were performed using one-way ANOVA and Spearman correlation.

**Results:**

Although tumor stem cell properties were confirmed in GSC cultures, their in vitro and in vivo morphology and behavior displayed considerable tumor-to-tumor variability. Drug screening revealed significant differences in the sensitivity to anticancer drugs (*p* < 0.0001). The patient-specific vulnerabilities to anticancer drugs displayed a heterogeneous pattern. They represented a variety of mechanistic drug classes, including apoptotic modulators, conventional chemotherapies, and inhibitors of histone deacetylases, heat shock proteins, proteasomes and different kinases. However, the individual GSC cultures displayed high biological consistency in drug sensitivity patterns within a class of drugs. An independent laboratory confirmed individual drug responses.

**Conclusions:**

This study demonstrates that patient-derived and treatment-naïve GSC cultures maintain patient-specific traits and display intertumoral heterogeneity in drug sensitivity to anticancer drugs. The heterogeneity in patient-specific drug responses highlights the difficulty in applying targeted treatment strategies at the population level to GBM patients. However, HTS can be applied to uncover patient-specific drug sensitivities for functional precision medicine.

**Electronic supplementary material:**

The online version of this article (10.1186/s12885-019-5861-4) contains supplementary material, which is available to authorized users.

## Background

Glioblastoma (GBM) is a devastating form of cancer. Unselected patients have a median survival time of less than one year, which increases to ~ 15 months in patients eligible for surgery, radiation and chemotherapy [1]. Despite a range of therapeutic approaches, little improvement has been gained over the recent decades [2].

The lack of therapeutic progress may be attributed to the complex cellular and molecular heterogeneity in GBM, both between patients [3, 4] and within individual tumors [5, 6]. Despite the heterogeneity of the disease, current treatment modalities are standardized to all patients, and clinical trials largely investigate treatment effects at the population level [7–9]. However, early phase trials of targeted therapies commonly report single or a few responders although they fail to demonstrate a survival benefit in the overall trial cohort [2, 10, 11]. These clinical response patterns suggest the presence of heterogeneity in the sensitivity to anticancer drugs; however, how tumor heterogeneity is reflected in individual drug sensitivity patterns in the treatment-naïve disease has not been established.

At the cellular level, a subpopulation of GBM cells, glioblastoma stem cells (GSCs), represents the top of a proliferative hierarchy in GBM. These cells can reconstruct the entire cellular spectrum in GBM, and give rise to highly infiltrative tumor growth in serial xenotransplantation [12]. As GSCs experimentally confer resistance to radiation and chemotherapy, these cells are presumed to be the cause of the inevitable tumor relapse [12]. We and others [13–17] have previously shown that upon propagation, patient-derived GSCs maintain their ability to form invasive tumors, preserve individual tumor traits at the genetic and expression level, and maintain a range of individual clones, thus representing an individualized model of the parent tumor.

Preclinical drug discovery studies in GBM commonly follow the traditional format focusing in compounds that exhibit broad efficacy across several samples for further advancement to clinical investigation [18–21]. Considering the disappointing results of clinical trials exploring targeted treatments at the population level in GBM, we aimed to explore the individual variation of drug sensitivity patterns in low passage, patient-derived and treatment-naïve GSCs to a large panel of anticancer drugs using automated high-throughput screening (HTS) and drug sensitivity scoring. We further investigated biological consistency and reproducibility of drug sensitivities to evaluate whether drug sensitivity and resistance testing (DSRT) using HTS can be translated to a clinical setting for functional precision medicine.

## Methods

### Cell cultures

Glioblastoma biopsies were obtained from 12 informed patients with explicit written consent undergoing surgery for GBM at Oslo University Hospital, Norway as approved by The Norwegian Regional Committee for Medical Research Ethics (REK 2017/167). The GSC cultures were established both from several focal tumor biopsies and ultrasonic aspirate generated during surgery. The IDH status was evaluated by immunohistochemistry and sequencing, and the MGMT promoter methylation status was evaluated by methylation-specific quantitative PCR. Cell cultures were established and maintained in serum-free media containing bFGF and EGF (both R&D Systems), as previously described [14]. Differentiation was induced, and cells fixed and stained, as previously described [14]. Images were acquired using Olympus Soft Imaging Xcellence software v.1.1. The total number of cells from one passage to the next in serial passages was extrapolated using the formula (total number of cells from previous passage/cells plated) x (total number of cells from current passage). All experiments in this study have been performed within the 10th passage of individual GSC cultures. Patient characteristics are summarized in Additional file 1.

### Flow cytometry analysis

Cells were suspended in PBS with 2% fetal bovine serum (Biochrom) and stained with directly conjugated antibodies (CD15-PerCP, R&D Systems, CD44-APC, Thermo Fisher Scientific, CD133-PE, Miltenyi Biotec, CXCR4-PE, Miltenyi Biotec) according to the manufacturer’s instructions. Cells were washed three times before analysis by flow cytometer LSRII (BD Bioscience). FlowJo software v.10.4.1 was used for data analysis. Dead cells were identified by propidium iodine (Thermo Fisher Scientific), and doublets were excluded by gating.

### Intracranial transplantation

The National Animal Research Authority approved all animal procedures (FOTS 8318). C.B.-17 SCID female mice (7–9 weeks old, Taconic) were anesthetized with an injection of zolazepam (3.3 mg/mL), tiletamine (3.3 mg/mL), xylazine (0.45 mg/mL) and fentanyl (2.6 μg/mL) and placed in a stereotactic frame (David Kopf Instruments). Cells were prepared and transplanted, as previously described [14]. The animals were regularly monitored for signs of distress and killed by cervical dislocation after 15 weeks or earlier if weight loss > 15% or neurological symptoms developed. The brains were harvested and further processed as previously described [14]. Images of brain sections were acquired using Axio Scan.Z1 (Carl Zeiss). Processing of images was performed using ImageJ 2.0.

### Drug collection and drug sensitivity and resistance testing

The oncology drug collection consisted of 461 compounds and covered most U.S. Food and Drug Administration and European Medicines Agency (FDA/EMA)-approved anticancer drugs and investigational compounds with a broad range of molecular targets. The complete drug collection is listed in Additional file 2. The compounds were dissolved in 100% dimethyl sulfoxide (DMSO) and dispensed on 384-well plates using an acoustic liquid handling device, Echo 550 (Labcyte Inc). The pre-drugged plates were kept in pressurized Storage Pods (Roylan Developments Ltd.) under inert nitrogen gas until needed. The patient-derived GSCs were plated at a density of 3000 cells/well using a MultiDrop Combat (Thermo Scientific) peristaltic dispenser. The plates were incubated in a humidified environment at 37 °C and 5% CO_2_, and after 72 h cell viability was measured using CellTiter-Glo® Luminescent Cell Viability Assay (Promega) with a Molecular Device Paradigm plate reader. The resulting data were normalized to negative control (DMSO) and positive control wells (benzethonium chloride). The quantification of drug sensitivity was utilized by the drug sensitivity score (DSS), as previously described [22, 23]. In brief, each drug was evaluated over a 5-point dose-escalating pattern covering the therapeutic range. The resulting dose-response was analyzed by automated curve fitting defined by the top and bottom asymptote, the slope, and the inflection point (EC_50_). The curve fitting parameters were used to calculate the area defined as area of drug activity (between the 10 and 100% relative inhibition to positive and negative control) into a single measure as the DSS. The selective drug sensitivity score (sDSS) of each compound was calculated as the difference between the DSS in the individual culture and the average DSS of all screened GBM cultures. One culture (T1505) was excluded from the analysis of the overall drug sensitivity due to an error in the automatic seeding procedure for 29% (132/461) of the drug responses.

### Validation experiments

Cells were plated at 5000 cells/well in a 96-well plate (Sarstedt, Germany) under sphere conditions, cultured for 24 h before the addition of drugs and further incubated for 72 h. Viability was assessed using Cell Proliferation Kit II XTT (Roche) solution incubated for 24 h before analysis on a PerkinElmer EnVision. The viability is corrected for the background signal and reported relative to negative control (DMSO), as the mean and standard error to the mean of five independent experiments.

### Gene expression analysis

Next generation sequencing and gene expression microarray experiments were performed at the Genomics and Bioinformatics Core Facility at the Norwegian Radium Hospital, Oslo University Hospital (Norway). The library preparation for RNA sequencing was performed using the Truseq mRNA Illumina protocol, and the samples were sequenced on the Illumina HiSeq platform (paired end 2 × 75 bp). Normalized expression data was further analyzed in J-Express 2011. Subgrouping of the GSC cultures as proneural or mesenchymal was performed by analyzing gene expression microarray data using the HumanHT-12 chip (Illumina). Unsupervised hierarchical clustering was performed according to the gene panels described by Mao et al. and Phillips et al. [24, 25]. Quality issues led to one culture (T1461) not being successfully sequenced and could not be included in the gene expression analyses.

### Statistical considerations

Data analysis and graphic presentation were undertaken using GraphPad Prism 7.0, J-Express 2012 (Molmine), Microsoft Excel 14.7.3 and R. Correspondence analyses and evaluation of the GSC culture subgrouping were performed using J-Express 2012. Unsupervised hierarchical clustering and heat maps were generated using J-Express 2012, GraphPad Prism 7.0, and R. Statistical analysis of the overall drug sensitivity between cultures was performed using non-parametric one-way ANOVA of ranks with Kruskal-Wallis test. Correction for multiple comparisons was done by Dunn’s test. The correlation analyses were performed using Spearman correlation (ρ). A *p*-value < 0.05 was considered significant.

## Results

### Intertumoral heterogeneity in patient-derived GSC cultures

The robustness of the patient-derived GSC model system in preserving the tumorigenicity and molecular features of the parent tumor is well documented by us and others [12–16, 26]. Such patient-derived GSCs, however, display considerable intertumoral differences in morphology and behavior in vitro and in vivo [12, 14].

**Fig. 1:**
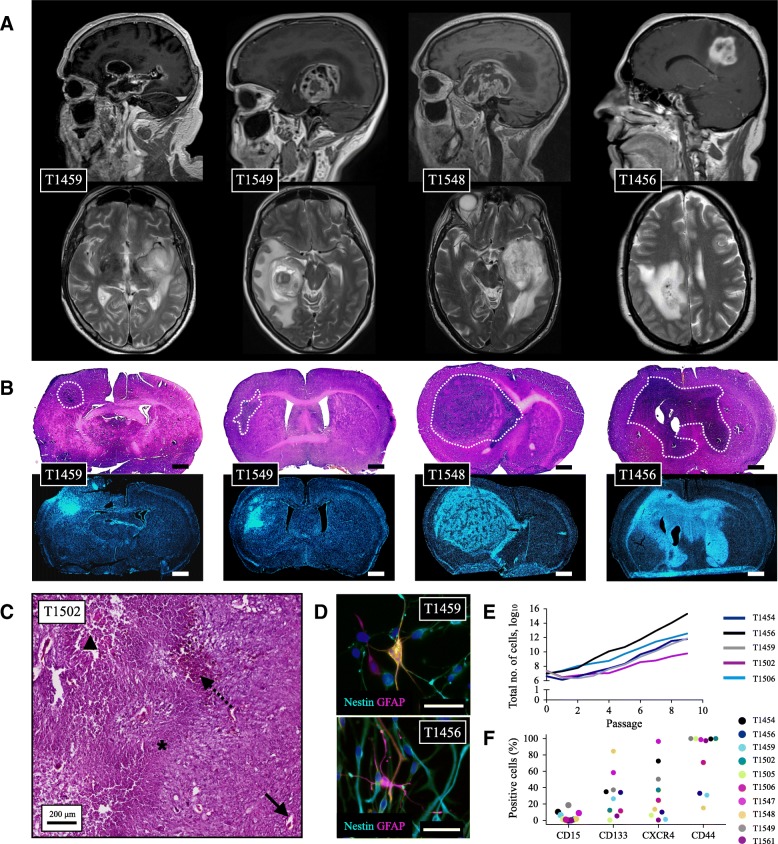
Characterization of patient-derived GSCs. Magnetic resonance imaging of four GBMs in the study cohort (**a**) and the corresponding xenografts (**b**) demonstrating that GSC cultures established from a heterogeneous GBM population display culture-to-culture heterogeneity in their in vivo formation characteristics. Images in (**b**) are stained with Hematoxylin & Eosin (**h**&**e**) in the upper image and Hoechst 33258 in the lower image. Tumor borders are macro-anatomically delineated. Scale bar 1 mm. (**c**) All histopathological features of glioblastoma were identified, including pathological angiogenesis (whole arrow), intratumoral hemorrhages (dotted arrow), tumor necrosis (triangle), pseudopalisading (asterisk) and nuclear atypia with aberrant mitoses. All tumors were xenografted to ≥2 mice. (**d**) Upon differentiation, the cells displayed a more mature morphology and stained positive for nestin and GFAP, however the individual GSC culture displayed intertumoral variability in their differentiation morphology. Scale bar 50 μm. (**e**) The cultures displayed variability in their capacity for total cell yield following serial passages, and (**f**) intertumoral heterogeneity in expression of stem cell markers (**f**). Expression of stem cell markers are data generated from *n* = 1 experiments in the individual cultures

In this sample cohort, eleven cultures formed free-floating tumorspheres, while one culture proliferated adherently (T1505). The individual cultures maintained their morphology upon serial passages and could be serially expanded. Intertumoral differences were observed in the in vitro spheroid and differentiation morphology, expression of GSC markers, total cell yield after serial passaging, and in vivo tumor formation characteristics (Fig. 1). Overall, the GSC cultures presented with considerable tumor-to-tumor variability in both morphology and behavior in vitro and in vivo, while maintaining culture specific characteristics.

### Intertumoral heterogeneity in drug sensitivity to anticancer drugs

Subsequently, we explored whether the intertumoral heterogeneity among GSC cultures is reflected in the sensitivity to a collection of 461 anticancer compounds using automated high-throughput technology. An overview of the drug collection is provided in Table 1. Reproducibility of the HTS was assessed by repeated screenings evaluated by a blinded investigator and displayed a ranked correlation of r = 0.823 (Spearman, *p* < 0.0001). The median passage number at the time of drug screening was 3 (range: 1–7).

**Table 1:** Overview of drug collection

Drug class	Approved	Investigational (Phase I-III)	Preclinical	Total
Conventional chemotherapy	58	5	3	66
Kinase inhibitor	32	172	26	230
Rapalog	4	1	0	5
Immunomodulatory	10	3	0	13
Differentiating/epigenetic modifier	10	21	20	51
Hormone therapy	18	3	1	22
Apoptotic modulator	0	12	3	15
Metabolic modifier	8	5	4	17
Kinesin inhibitor	0	3	0	3
NSAID	2	0	0	2
Heat shock protein inhibitor	0	6	2	8
Proteasome inhibitor	2	1	1	4
Hedgehog inhibitor	1	1	0	2
Other	7	8	8	23
Total number (% of total)	152 (33%)	241 (52%)	68 (15%)	461

**Fig. 2:**
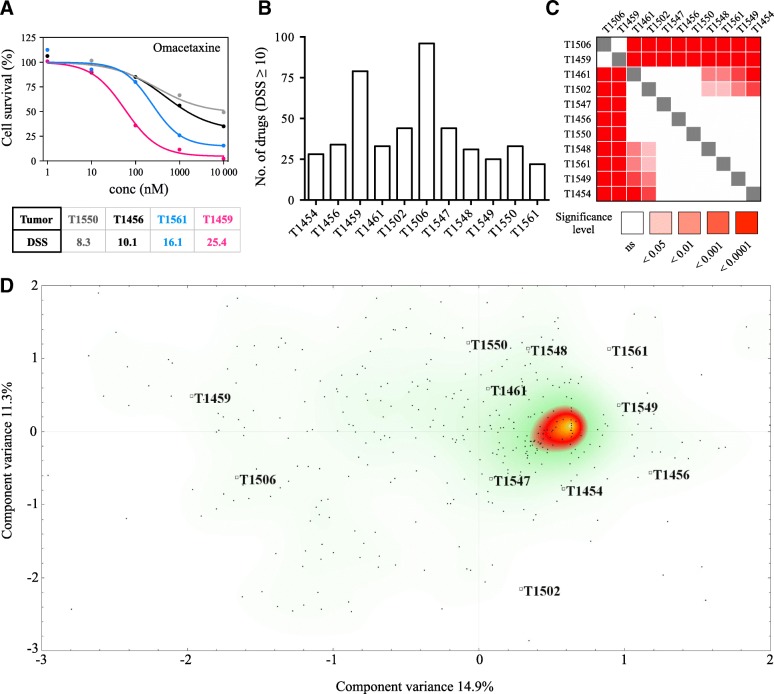
GSC sensitivity to anticancer drugs. (**a**) Presentation of four drug responses from the DSRT to the FDA-approved protein synthesis inhibitor omacetaxine. The dose-response curves and DSS demonstrate a drug response below the threshold defined as moderate activity (DSS ≥10) and three other responses with increasing efficacy from moderate to very strong. (**b**) Number of drugs from the DSRT in each GSC culture with a DSS ≥10. (**c**) Significance table of the distribution of the overall drug sensitivity to the drug collection (*n* = 461 drugs) in the primary GSC cultures. Using a non-parametric one-way ANOVA of ranks corrected for multiple comparisons, a significant difference was observed in the overall drug sensitivity (*p* < 0.0001). (**d**) Correspondence analysis of all drug responses displays a clear separation of the two most sensitive cultures along the first component variance (14.9%), whereas no identified pattern explained the spread of the cultures along the second component variance (11.3%). Each dot in the scatter plot represents individual drugs (rows), while individual tumors are highlighted (columns)

A DSS ≥10 was defined as the threshold to classify a drug response as moderate to strong (Fig. 2a). Following DSRT, in total, 115 compounds (25% of the entire drug collection) displayed this response in the GSC culture cohort. The median was 33 drugs (range: 22–95). Two cultures, T1459 and T1506, clearly had higher number of drugs with a DSS ≥10, 79 and 95 drugs, respectively (Fig. 2b). The sensitivity to any given drug was, however, heterogeneous, as 93 of the 115 drugs (81%) with a DSS ≥10 displayed intersample differences equivalent to a moderate to strong difference in sensitivity (∆DSS ≥10, DSS_max_ - DSS_min_). The overall sensitivity to the entire drug collection (*n* = 461) significantly differed among all GSC cultures (p < 0.0001). Based on the differences in the overall drug sensitivity, the cultures were broadly clustered into three major categories of most (T1459 and T1506), moderate (T1461, T1502, T1547, T1456, T1550) and least (T1454, T1561, T1549, T1548) sensitive cultures (Fig. 2c, Additional file 3). Correspondence analysis of the DSS to all drugs clustered the two most sensitive cultures distinctively apart along the first component variance (14.9%), while the second component variance (11.3%) spread the cultures without identifying any clear pattern of clustering (Fig. 2d).

Based on global gene expression profiling, the clustering of the GSC cultures differed from the clustering according to drug sensitivity, as the two most sensitive cultures clustered separately. We found more similarities in the gene expression between cultures categorized as moderate and least sensitive (T1456, T1454, T1548) than related to their overall drug sensitivity (Additional file 4). Further exploring selected gene panels involved in general drug resistance, drug metabolism, GSC related, and glioblastoma related genes did not identify any shared expression pattern of the most sensitive cultures compared to the others (Additional file 5).

### Heterogeneity in the sensitivity to classes of anticancer drugs

**Fig. 3:**
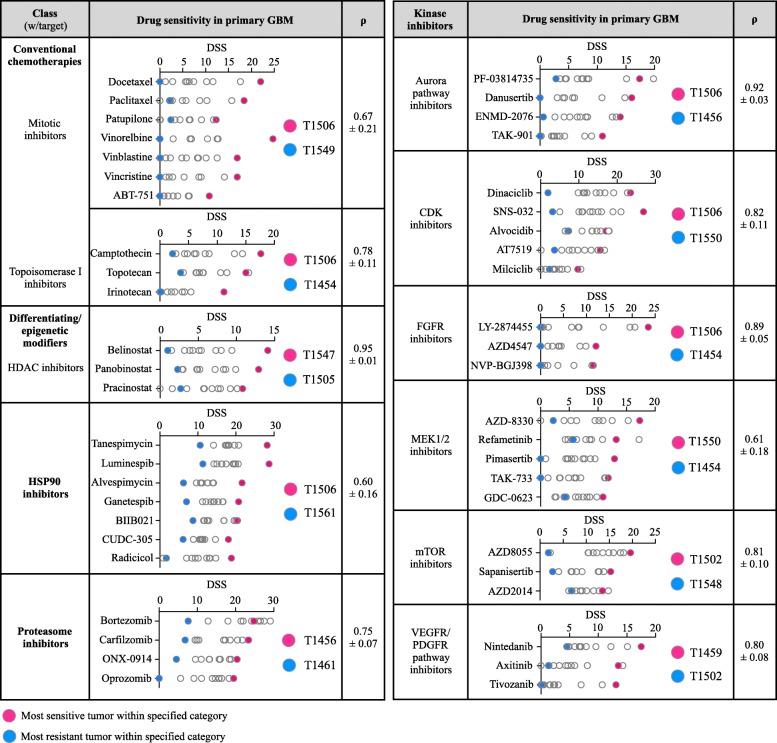
Drug sensitivity in primary GSCs across different drug classes and molecular targets. The figure displays drug class, the drug sensitivity in GSC cultures, and average (± SD) Spearman’s coefficient (ρ) from correlation matrices for drug categories that were represented with ≥3 drugs for the specific molecular target (*n* = 47 drugs in the figure, all drug sensitivity data in Additional file 3). Correlation matrices demonstrated that the sensitivity to a drug within a category was strongly associated with sensitivity to all other drugs within that drug category, demonstrating biological consistency and individual uniqueness in GSC cultures. Highlighted in red and blue are the tumors found with the highest and lowest sensitivity within the specified category, respectively

The overall drug sensitivity only explained a small proportion of the variance, suggesting that tumors can be grouped into a few subtypes. As 81% of the drugs with a DSS ≥10 also displayed ∆DSS ≥10 among all cultures, we explored how the heterogeneity in the sensitivity to anticancer drugs distributed across different mechanistic classes and molecular targets. The 115 drugs with a DSS ≥10 in any GSC culture represented a wide range of drug classes, including apoptotic modulators, conventional chemotherapies and inhibitors of histone deacetylases, heat shock proteins, proteasomes and different kinases. Across all classes and molecular targets, the distribution of drug sensitivities largely displayed a continuum from insensitive to the most sensitive tumor (Fig. 3).

To explore whether the GSC model system preserves the individual biological consistency of drug sensitivities, we categorized drug sensitivity patterns based on the specific molecular target within a class of drugs (e.g., MEK1/2 inhibitors in the kinase inhibitor class). We found a clear pattern in which drugs with a specific target displayed the highest efficacy in the same tumor. For instance, among MEK1/2 inhibitors with a DSS ≥10 (*n* = 5) in any GSC culture, T1550 was the most sensitive culture to four of five MEK1/2 inhibitors (and the 2nd most sensitive to the final inhibitor). Correlation matrices displayed that the average (±standard deviation) ranked correlation of the sensitivity to MEK1/2 inhibitors was 0.61 (±0.18) (Fig. 3). Similarly, the GSC cultures most resistant to a specific class of drug displayed a clear pattern of broad resistance to all drugs targeting the same specific molecular target. While being the most sensitive to MEK1/2 inhibitors, T1550 was the most resistant culture to CDK inhibitors (*n* = 5). The correlation matrices displayed that the average correlation of sensitivity to CDK inhibitors was 0.82 (±0.11) (Fig. 3). This consistency of individual drug sensitivity and resistance patterns was found across all major classes within the drug collection (Fig. 3). This demonstrated that individual biological traits involved in drug sensitivities are preserved and consistent in patient-derived GSC cultures and display individual uniqueness. In the DSRT, none of the GSC cultures displayed sensitivity to the standard-of-care, temozolomide (TMZ, Additional file 3).

### Validation of drug sensitivities

The heterogeneity of drug sensitivity patterns in individual GSC cultures demonstrated that DSRT could uncover patient-specific vulnerabilities and potential treatment options for functional precision medicine. However, for DSRT to guide decision-making in patient treatment, we investigated the manual reproducibility of selected compounds in an independent laboratory performed by different personnel. To obtain a closer description of the biologically relevant concentration range, we performed a narrower 5-point concentration range and defined reproducibility by the ability to capture the inflection range with similar levels of EC_50_-calculation and maximal inhibition. The independent validation confirmed the reproducibility by quantifying EC_50_ in similar low molar concentrations and reaching levels of maximal inhibition in different drugs across different tumors (Additional file 6).

### Taxonomy of GSCs based on drug sensitivity patterns

**Fig. 4:**
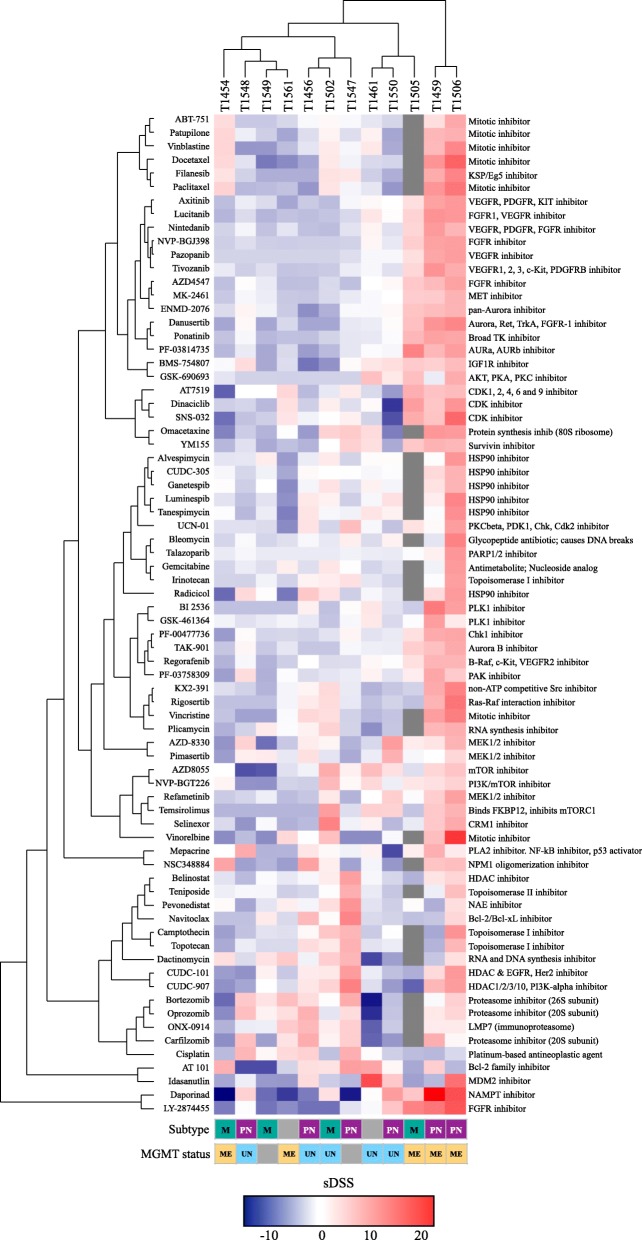
Unsupervised hierarchical clustering of drug sensitivity patterns in primary GBM and relation to subtype and MGMT status. Heat map and unsupervised hierarchical clustering of patient-specific drug responses (sDSS) with Euclidian distance (cultures and drugs). The heat map is filtered by DSS ≥10 and sDSS ≥ or ≤ 6.5 (*n* = 74 drugs). The two most sensitive cultures clustered separately and were both of a proneural subtype, with a methylated MGMT promoter. The four least sensitive cultures grouped together in the other major taxonomy; however, among the moderate and least sensitive cultures, no clear pattern was observed in the subtype classification or methylation status of the parent tumor. Even in the cultures clustering together, individual differences in sensitivities to different mechanistic classes of drugs were found (e.g., sensitivity to topoisomerase I inhibitors in T1459 compared to that in T1506, sensitivity to CDK-inhibitors in T1549 compared to that in T1561, sensitivity to mTOR-pathway inhibitors in T1456 compared to that in T1502, and sensitivity to MEK1/2 inhibitors in T1461 compared to that in T1550). Subtype; M: Mesenchymal, PN: proneural, gray box: not available data. MGMT promoter status: ME: Methylated MGMT promoter, UN: Unmethylated MGMT promoter, gray box: not available data

As the drug sensitivity and resistance patterns were linked to drug classes and molecular targets, we stratified the GSC cultures according to similar drug sensitivity patterns. For the stratification into patient-specific drug sensitivity for any given drug, we calculated the differential response in an individual culture from the average response in all GSC cultures. Thus, we quantified each drug response in each individual culture as either increased (+) or decreased (−), defining this as the selective DSS (sDSS) (Additional file 7). Correspondence analysis of the sDSS to all drugs clustered the cultures according to the overall sensitivity along the first component variance (19.1%), while the second component variance (12.8%) clustered the cultures based on the similarities in the sensitivity and resistance patterns (Additional file 7). Unsupervised hierarchical clustering revealed that the relationships among similar drug sensitivity patterns were based on the mechanistic target (Fig. 4, Additional files 8 and 9). The two most sensitive cultures were of the proneural subtype; however, in the moderate to least sensitive tumors, the proneural and mesenchymal subtypes were evenly interspersed (Fig. 4). The MGMT promoter methylation of the parent tumor status was not concordant with the clustering as the two most sensitive tumors and two of the four least sensitive tumors were MGMT promoter methylated.

**Fig. 5:**
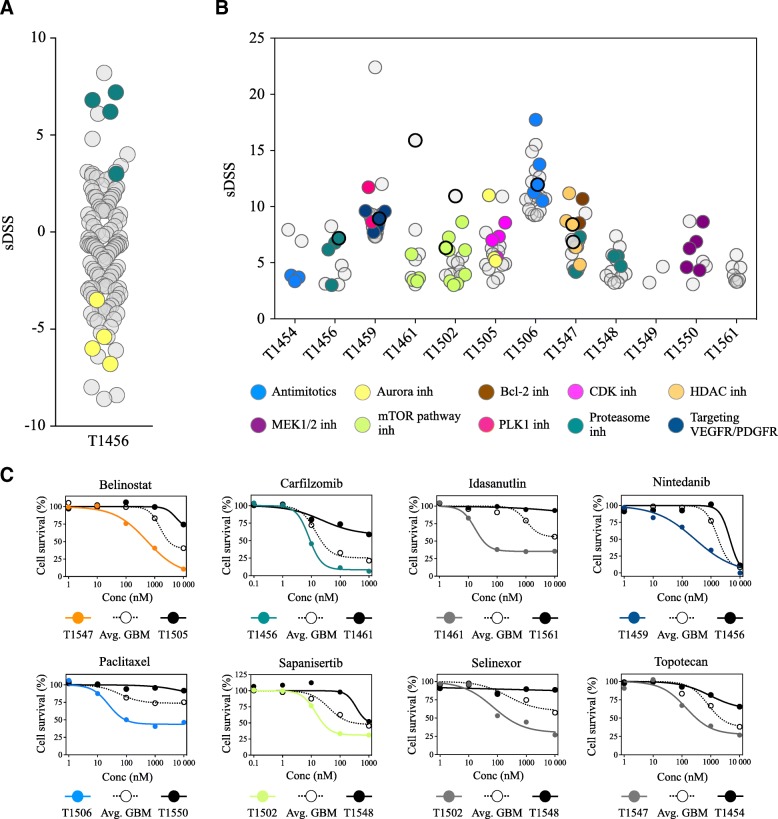
Heterogeneity in patient-specific drug responses in treatment-naïve GSCs. (**a**) Dot plot of the distribution of the patient-specific responses (sDSS) in T1456 to all drugs with DSS ≥10 in any GSC culture displays the enrichment of proteasome inhibitor (green) clustering with increased culture specificity and the insensitivity to aurora pathway inhibitors (yellow). (**b**) Dot plot displaying the distribution of the drug categories clustering with the highest patient-selectivity in individual GSC cultures. Drugs are filtered by DSS ≥10 and sDSS ≥3, and drug classes are filtered by O/E ≥ 3 for the individual culture. Classes of drugs enriched in individual cultures are highlighted and display the extensive intertumoral heterogeneity in patient-specific vulnerabilities to anticancer drugs. In cultures T1459, T1506 and T1547, the top 20 selective drug responses are presented. Of the drugs with DSS ≥10, three drugs singly target HDAC, whereas two drugs (CUDC-907 and CUDC-101) have dual targets by targeting HDAC along with PI3K or EGFR/Her2, respectively. In T1547, all five drugs that singly or as a dual target inhibit HDAC were found to have the highest patient selectivity and were highlighted within the category of HDAC inhibitors. For the PLK1 inhibitors and bcl-2 inhibitors, O/E was < 3 as only 2 drugs were represented in the drug collection; however, these drugs are highlighted as they displayed unique selectivity in T1459 and T1547, respectively. (**c**) Dose-response curves of selected drug responses displaying the most sensitive tumor (colored line, drug response is highlighted with enhanced rim in dot plot in B) and the least sensitive tumor (black line) compared to the average response in GBM (dashed line). All drugs have (i) been tested in clinical trials of GBM (nintedanib, paclitaxel, topotecan), (ii) are currently in clinical trials of GBM (belinostat (NCT02137759), sapanisertib (NCT02142803), and selinexor (NCT01986348), clinicaltrials.gov) or (iii) represent drugs within a class that are being investigated in GBM (carfilzomib; proteasome inhibitors, idasanutlin; mdm2 inhibitors, clinicaltrials.gov). Both insensitive and highly sensitive cultures are found in response to each drug

To comprehend the overall heterogeneity in drug sensitivities in the entire culture cohort, we calculated the enrichment of drugs with the same modes of action in individual cultures according to the ratio of observed versus expected (O/E, if expected number of drugs was < 1, the value was set to 1) (Fig. 5a). By selecting drugs that had at least moderate efficacy (DSS ≥10) increased patient-specificity (sDSS ≥3) and O/E ≥ 3 in individual cultures, we found eight different drug categories of various molecular targets to be enriched in the treatment-naïve GSC cultures (Fig. 5b). The stratification into patient-specific responses identified the GSC cultures with the highest vulnerability to any given drug or class of drug. The dose-response curves of drugs that have been investigated in clinical trials of GBM demonstrated the existence of both resistant and sensitive GSC cultures in the treatment-naïve disease (Fig. 5c). Similarly, drugs from various categories currently recruiting patients for trials in GBM displayed the same pattern including both existing resistant and sensitive GSC cultures in a heterogeneous GBM population (Fig. 5c).

## Discussion

This study demonstrates that treatment-naïve GSC cultures display individual morphological and behavioral traits in vitro and in vivo, and intertumoral heterogeneity in individual drug sensitivity patterns, reflecting biological diversity.

The variation in the sensitivity to anticancer drugs further describes the complexity of tumor heterogeneity in GBM. As each tumor is intricately heterogeneous, generalized treatment regimens are unlikely to substantially improve the survival of most GBM patients. Consistently, both early and late phase clinical trials investigating targeted therapies have not presented a survival benefit at the population level over previous decades [2, 7, 8]. Cases of responders are, however, commonly reported, which is indicative of patient heterogeneity in drug sensitivity [10, 11]. Biomarkers or subgrouping of patients have, unfortunately, not successfully categorized patients for stratified treatments.

Selection of patients for targeted treatment can be performed by genomics-based matching of GBMs to drug therapies. However, in glioma patients with druggable oncogenic mutations, individualized treatment decisions are difficult to apply clinically [27, 28], and in large investigational cohorts, the fraction of patients benefitting from genomic-based treatment decisions remains low [29, 30]. Consistently, a recent study exclusively recruited relapsed GBM patients with EGFR amplification to investigate the efficacy of dacomitinib (2nd generation pan-HER inhibitor). The authors reported limited activity in the trial cohort but noted a few responders without identifying biomarkers suggestive of response [11]. In vitro drug sensitivity testing offers a functional approach for precision medicine, by identifying patient-specific vulnerabilities to anticancer drugs. By utilizing DSRT for identification of patient-specific drug responses, the ex vivo HTS model system identifies GSC cultures that are especially vulnerable to a class of drug. The DSRT approach utilizing patient-specific drug sensitivities has been investigated in chemorefractory hematopoietic cancers, where linking ex vivo drug responses and molecular profiling achieved clinical remissions [22]. In a study conducted before the era of GSCs, 40 primary GBM patients were treated based on the results of in vitro drug sensitivity testing [31]. Despite the establishment of cultures that are less likely to represent the tumor of origin [13], the authors presented promising overall survival with a median of 20.5 months. Unfortunately, this study did not lead to further clinical trials; thus, whether drug sensitivity and resistance testing results in clinically useful treatment decisions in GBM is unclear.

Recently, drug discovery studies have utilized drug screening strategies of GBM biopsies cultured in serum-free media. These studies commonly follow the traditional format of drug discovery and primarily highlight broadly effective compounds that demonstrate antitumor activity across several cultures in vitro [19, 32] and in vivo [20, 21]. In contrast, and to address the well-established tumor heterogeneity in GBM, we focused on how the individual variation in drug sensitivities is distributed in the treatment-naïve disease. This resulted in an important finding of the existence of drug resistant GSC cultures within all drug categories. This has implications for preclinical GBM research following the traditional format, as generalizing findings of therapeutic efficacy generated from a few selected GBM cultures has limited translational value in a heterogeneous GBM population.

Two recent studies have added complexity to individualized therapy options using drug screening strategies [33, 34]. After generating different clones from the same tumor, the authors found clone-by-clone differences in individual drug sensitivities. To maximize the clonal diversity in the individual GSC cultures, we established cultures from several focal biopsies and tumor aspirates generated from surgical ultrasonication. While the GSC culture system can maintain diverse individual clones from the same tumor [17], it is important to consider that these cultures represent a subpopulation of the total clonal variation, underestimating the complexity of drug responses. In addition, as we evaluated drug sensitivity at the culture level, clone-by-clone differences are not uncovered.

We found that drugs from different mechanistic classes displayed patient-specific activity (sDSS) in different GSC cultures. Thus, selecting generalized treatment options appears difficult as most drugs displayed a wide range of efficacy. Drugs from different mechanistic classes, e.g., the kinase inhibitor nintedanib, the antimitotic paclitaxel, the rapalog temsirolimus and the topoisomerase I inhibitor topotecan, demonstrated a moderate to strong response in a few cultures. These findings mirror the situation in early phase trials of GBM in which the clinical investigation of nintedanib, paclitaxel, temsirolimus and topotecan in GBM have all resulted in an overall negative efficacy, while a few or a minor subgroup of responders is observed [35–38].

We found a uniform resistance to TMZ in the DSRT, despite several of the cultures being obtained from MGMT-methylated tumors. The setup of the DSRT could explain this, as the evaluation of cell viability was performed after 72 h of incubation. In accordance with previous reports by us and others [20, 39–41], evaluation of sensitivity to TMZ using clinical relevant drug concentrations requires longer incubation than 72 h in cell viability assays. Drugs that potentially would benefit from a longer incubation time due to their mode of action could potentially turn out as false negative using a HTS platform. The time-point of effect evaluation, however, was based on a broad evaluation of the whole drug collection as well as data from other cell types [22].

Since the first report of tumor cells with stem cell properties in GBM, the GSC model system has been well-recognized as a superior representation of the disease compared to established cell lines cultured in serum-containing media [13, 42]. Due to the strength of patient-derived GSCs in retaining the key characteristics of the parent tumor and in vivo behavior resembling GBM, individualized GSC cultures represent a patient-specific model of the tumor, with the possibility for individualized therapy strategies [43]. However, we acknowledge the inherent limitation in using patient-derived GSCs enriched in vitro as a model for drug discovery as important aspects of the in vivo GBM biology, including blood-brain barrier, tumor microenvironmental and immunomodulatory involvement in tumor progression and therapeutic resistance, are not addressed. Despite these drawbacks, a growing body of evidence highlights the clinical importance of targeting GSCs to improve therapy as a GSC gene signature, propagation of GSCs in vitro, and the in vitro sensitivity to TMZ are independent predictors of patient outcome [44–46]. To reflect the uniqueness of individual GBMs, we used low passage primary cultures from 12 different treatment-naïve primary IDH^wt^ GBM patients, which were sampled and cultured to maintain clonal diversity within each tumor. In addition, the biological reproducibility of selected drug sensitivities demonstrates consistency in HTS results for translation of DSRT to the patient bedside for individualized therapy.

## Conclusions

In summary, we have shown that individualized GSC cultures display an extensive intertumoral heterogeneity in sensitivity to anticancer drugs, which mirrors the clinical situation in early-phase trials of GBM. As patient-specific drug sensitivities are represented from a range of anticancer drugs with different modes of action, the intertumoral heterogeneity of individual drug sensitivities reflects the difficulty in applying targeted treatment strategies at the population level in GBM. We will further pursue the ability to translate our drug screening strategy to the patient bedside for functional precision medicine and individualized therapy.

## Additional files

Additional file 1: *Patient characteristics*. Patient characteristics of which all patient-derived GSC cultures were obtained. (XLSX 57 kb)

Additional file 2: *Drug collection*. The drug collection used in this study (XLSX 73 kb)

Additional file 3: *Drug sensitivity scores.* Complete data set of the drug sensitivity score generated in this study (XLSX 75 kb)

Additional file 4: *Global gene expression analyses.* (A) Correspondence analysis of global gene expression data displayed a tumor distribution contrasting the overall drug sensitivity analyses with no clear separation of the two most sensitive tumors from the others. Each dot in the scatter plot represents individual genes (rows), while individual tumors are highlighted (columns). (B) Unsupervised hierarchical clustering with distance matrix (average linkage, Pearson correlation). (PDF 657 kb)

Additional file 5: *Gene expression analyses of GSC cultures related to selected genes of drug resistance, metabolism, GSC- and GBM genes.* Unsupervised hierarchical clustering of expressed genes related to (A) drug resistance, (B) drug metabolism, (C) GSCs, and (D) GBM. In all analyses of selected gene panels, the clusters do not separate the most sensitive tumors from the others. Scale bar in all heat maps: log2-values. The cultures highlighted in red text were the two most sensitive GSC cultures from the drug screening. (PDF 289 kb)

Additional file 6: *Validation of selected compounds from the drug screening.* We identified drugs with a high DSS and increased patient-specificity (sDSS) and verified the pattern of drug responses in an independent laboratory. (A-C) T1454, (D-F) T1456, and (G-I) T1459. The dose-response curves in the validation experiments are calculated from the mean ± standard error of the mean in five independent experiments and fitted on the basis of a four-parameter sigmoidal logistic fit function. (PDF 342 kb)

Additional file 7: *Calculation of sDSS, distribution and correspondence analysis of sDSS from the DSRT.* (A) Dose-response curves to bortezomib in GSC cultures ranging from the least sensitive tumor (upper curve, T1461) with a DSS of 7.6 to the most sensitive tumor (T1547, lower curve) with a DSS of 29.1. Average DSS across all cultures is highlighted in blue. (B) By using the average DSS in all GBM as a reference, the cultures were classified according to the relative increased or decreased sensitivity to bortezomib presented as selective DSS (sDSS) in the waterfall plot. (C) Distribution of sDSS of the entire drug collection significantly differed among the cultures (*p* < 0.0001) (one-way ANOVA corrected for multiple comparisons, Kruskal-Wallis test with Dunn’s multiple comparisons test), and the GSC cultures broadly clustered into three categories. (D) Correspondence analysis of sDSS separated the cultures into most, moderate and least sensitive along the first (component variance 19.1%), while the second component variance (component variance 12.8%) identified the patterns of similar drug sensitivities according to the drug category. Each dot in the scatter plot represents individual drugs (rows), while individual tumors are highlighted (columns). (PDF 214 kb)

Additional file 8: *Heat map of DSS in all drugs.* Heat map and unsupervised hierarchical clustering of absolute effects (DSS) of the entire drug collection. Gray: failed/missing drug response. (PDF 148 kb)

Additional file 9: *Heat map of sDSS in all drugs*. Heat map and unsupervised hierarchical clustering of relative effects (sDSS) of the entire drug collection. Gray: failed/missing drug response. (PDF 148 kb)

## Data Availability

Data from the drug screening are included in this published article and its additional files. All other data used in the current study are available from the corresponding author on reasonable request.

## Abbreviations

CDK: Cyclin-dependent kinase
DSRT: Drug sensitivity and resistance testing
DSS: Drug sensitivity score
GBM: Glioblastoma
GSC: Glioblastoma stem cell
HTS: High-throughput screening
IDH: Isocitrate dehydrogenase
MEK: Mitogen activated protein kinase
MGMT: O^6^-methylguanine–DNA methyltransferase
sDSS: Selective drug sensitivity score
TMZ: Temozolomide
