# Supplementary figures and images for: Intertumoral heterogeneity in patient-specific drug sensitivities in treatment-naïve glioblastoma
Source: BMC Cancer. 2019 Jun 25;19:628. doi: 10.1186/s12885-019-5861-4 (PMC6593575; doi:10.1186/s12885-019-5861-4)

A

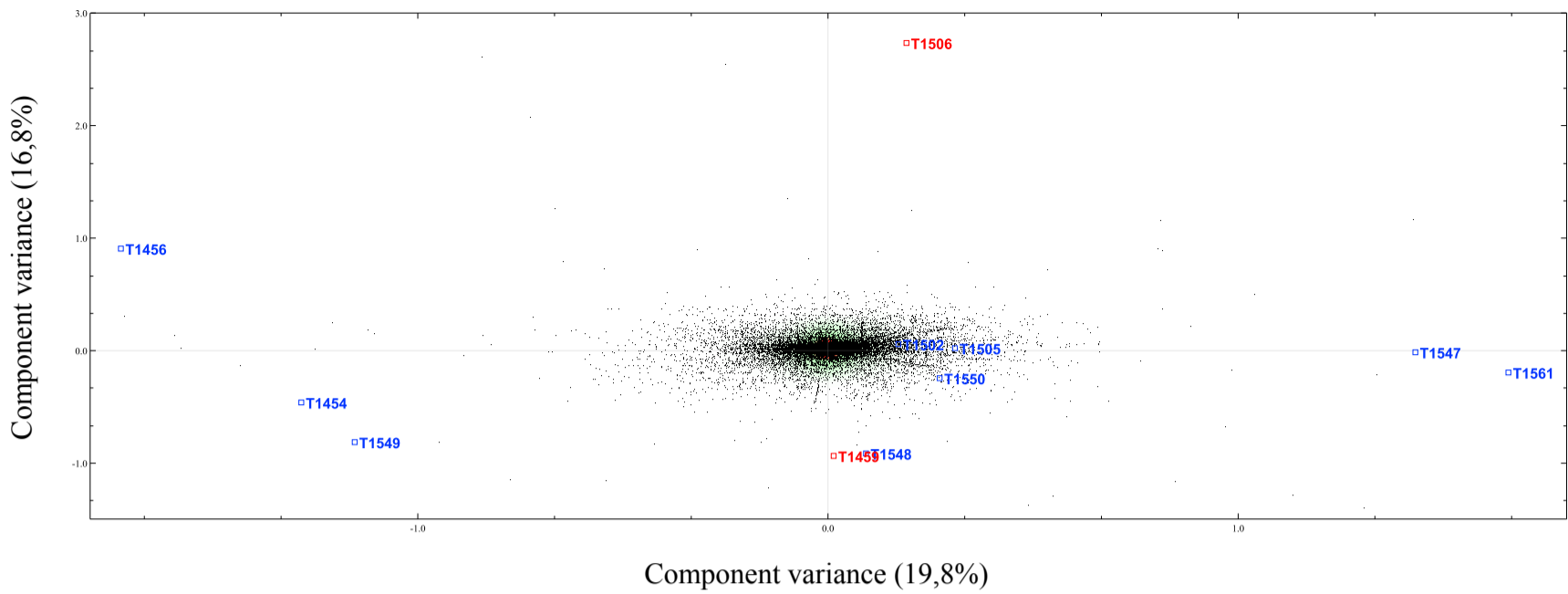

B

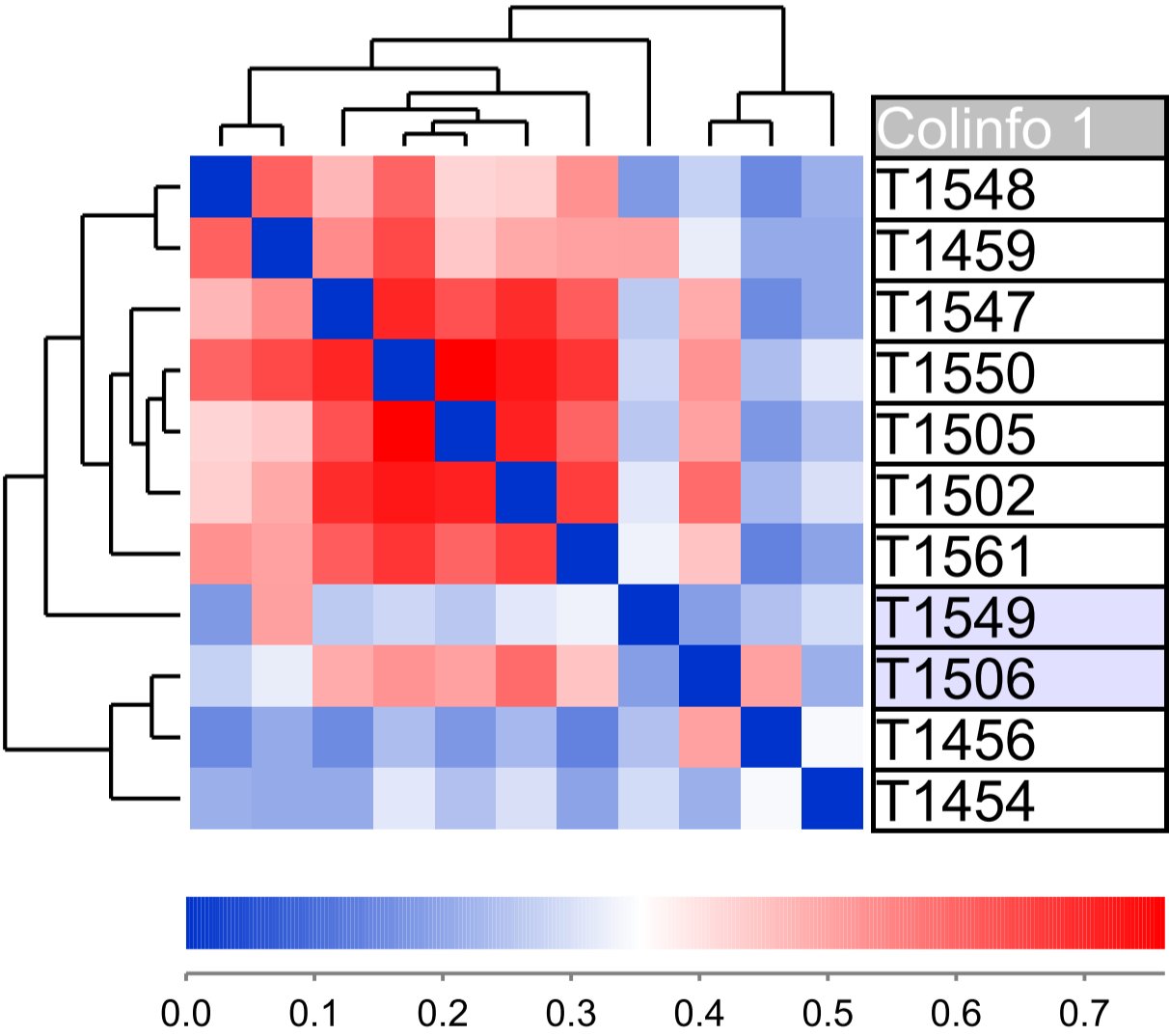

Supplement: Supplementary file 4 — Global gene expression analyses. (A) Correspondence analysis of global gene expression data displayed a tumor distribution contrasting the overall drug sensitivity analyses with no clear separation of the two most sensitive tumors from the others. Each dot in the scatter plot represents individual genes (rows), while individual tumors are highlighted (columns). (B) Unsupervised hierarchical clustering with distance matrix (average linkage, Pearson correlation). (PDF 657 kb) [file 12885_2019_5861_MOESM4_ESM.pdf]

A

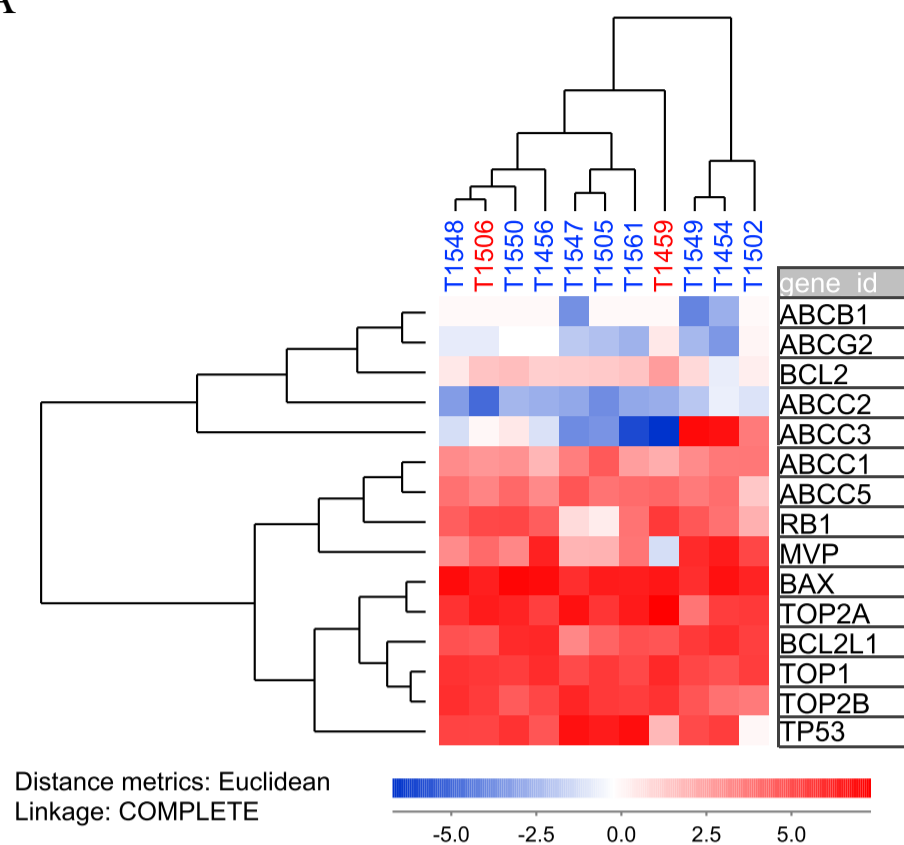

B

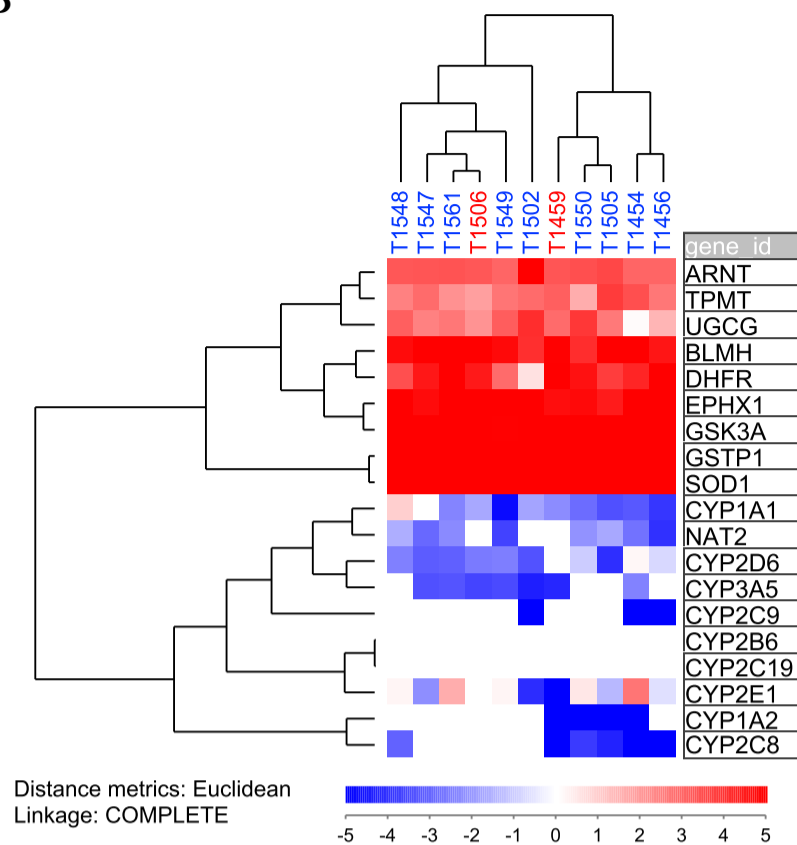

C

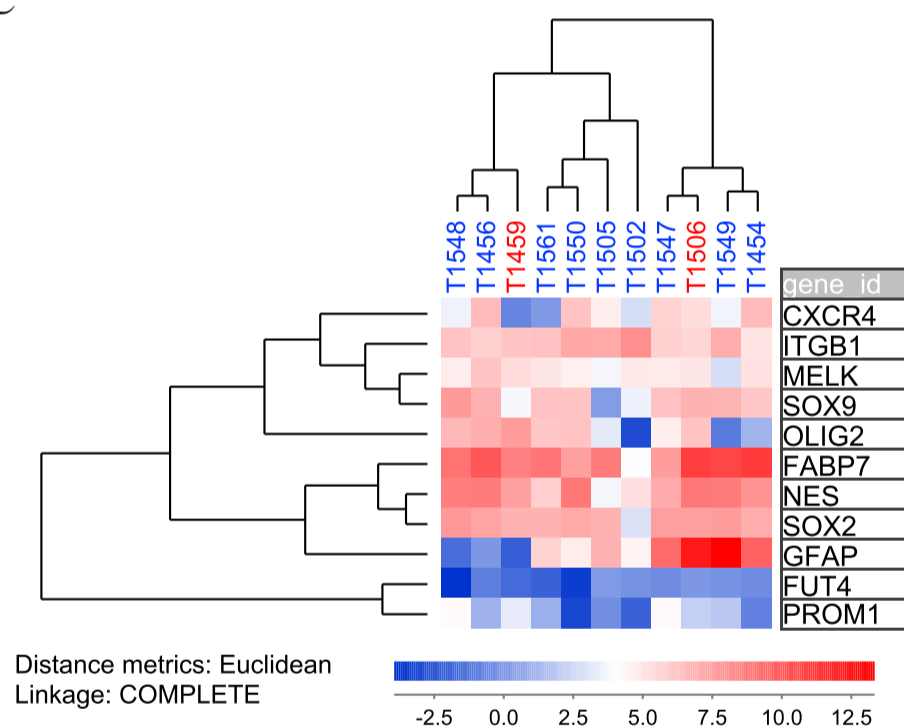

D

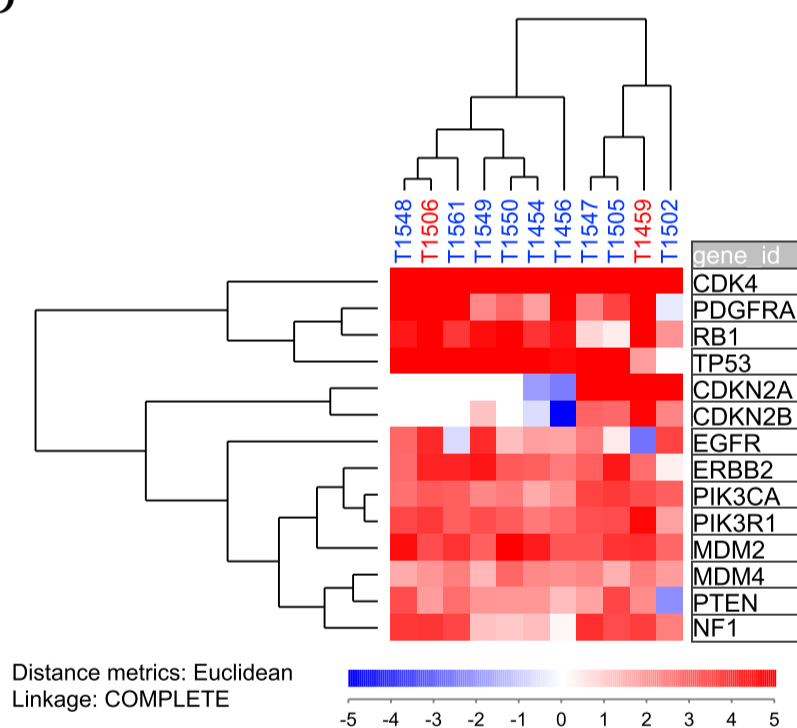

Supplement: Supplementary file 5 — Gene expression analyses of GSC cultures related to selected genes of drug resistance, metabolism, GSC- and GBM genes. Unsupervised hierarchical clustering of expressed genes related to (A) drug resistance, (B) drug metabolism, (C) GSCs, and (D) GBM. In all analyses of selected gene panels, the clusters do not separate the most sensitive tumors from the others. Scale bar in all heat maps: log2-values. The cultures highlighted in red text were the two most sensitive GSC cultures from the drug screening. (PDF 289 kb) [file 12885_2019_5861_MOESM5_ESM.pdf]

A

Bortezomib

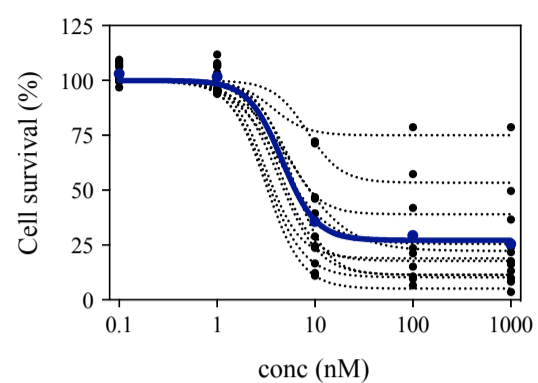

B

Bortezomib

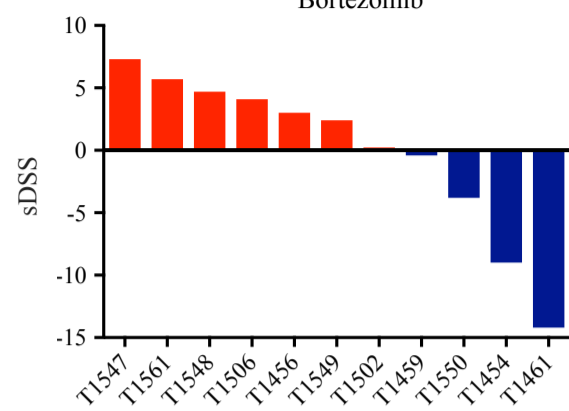

C

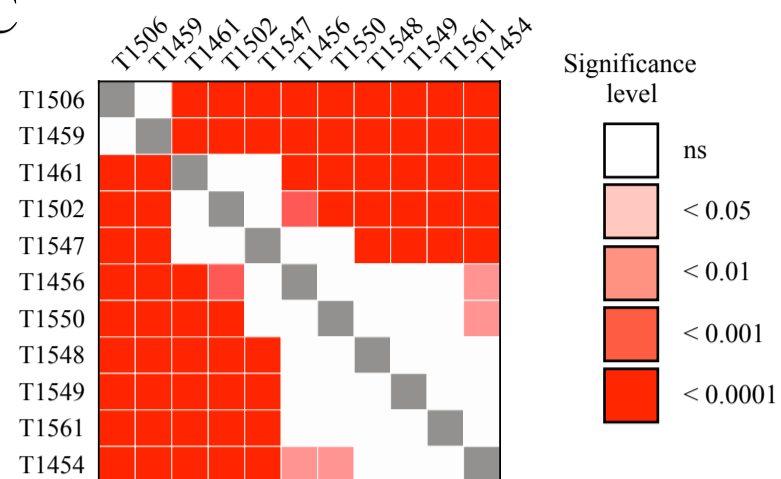

D

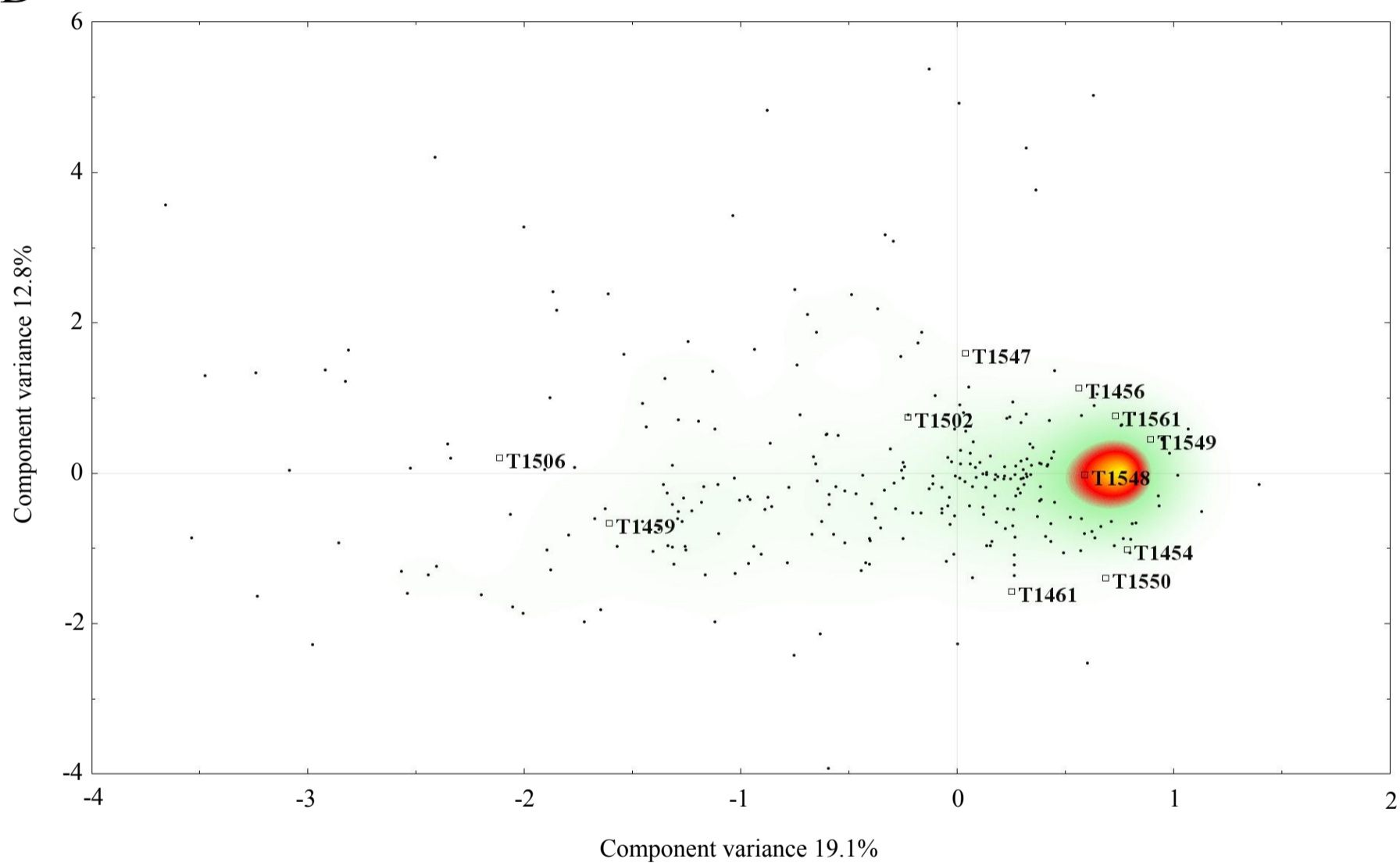

Supplement: Supplementary file 7 — Calculation of sDSS, distribution and correspondence analysis of sDSS from the DSRT. (A) Dose-response curves to bortezomib in GSC cultures ranging from the least sensitive tumor (upper curve, T1461) with a DSS of 7.6 to the most sensitive tumor (T1547, lower curve) with a DSS of 29.1. Average DSS across all cultures is highlighted in blue. (B) By using the average DSS in all GBM as a reference, the cultures were classified according to the relative increased or decreased sensitivity to bortezomib presented as selective DSS (sDSS) in the waterfall plot. (C) Distribution of sDSS of the entire drug collection significantly differed among the cultures (p < 0.0001) (one-way ANOVA corrected for multiple comparisons, Kruskal-Wallis test with Dunn’s multiple comparisons test), and the GSC cultures broadly clustered into three categories. (D) Correspondence analysis of sDSS separated the cultures into most, moderate and least sensitive along the first (component variance 19.1%), while the second component variance (component variance 12.8%) identified the patterns of similar drug sensitivities according to the drug category. Each dot in the scatter plot represents individual drugs (rows), while individual tumors are highlighted (columns). (PDF 214 kb) [file 12885_2019_5861_MOESM7_ESM.pdf]

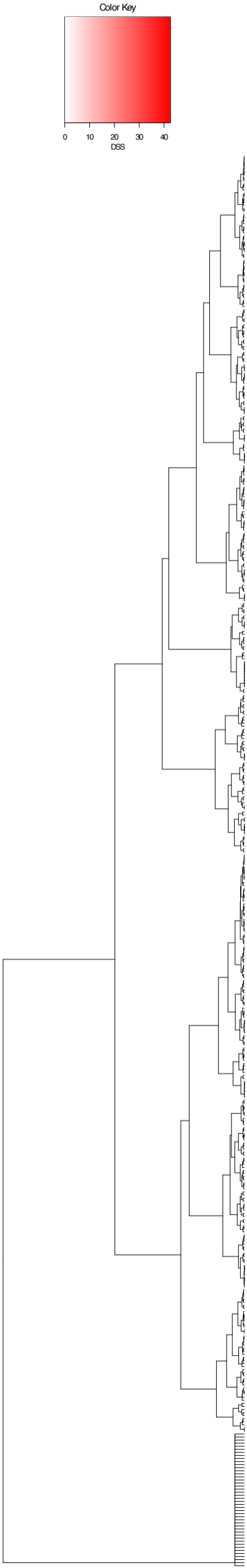

CTG DSS Heatmap 461 Drugs  
(rdist=spearman and rclust = ward, cdist = manhattan and cclust = ward)

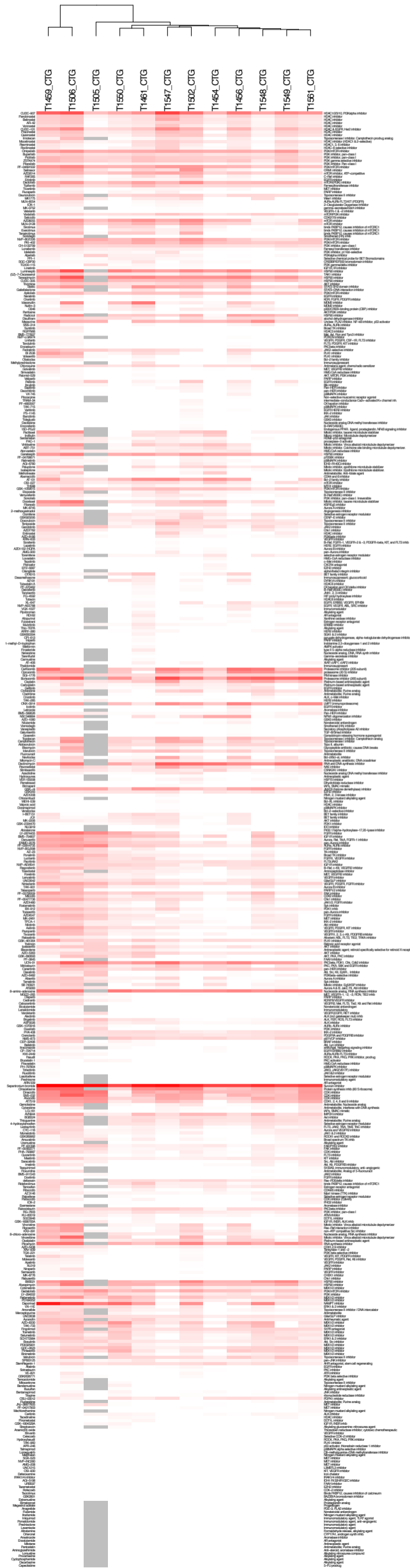

Supplement: Supplementary file 8 — Heat map of DSS in all drugs. Heat map and unsupervised hierarchical clustering of absolute effects (DSS) of the entire drug collection. Gray: failed/missing drug response. (PDF 148 kb) [file 12885_2019_5861_MOESM8_ESM.pdf]
