# Supplementary material for: Intertumoral heterogeneity in patient-specific drug sensitivities in treatment-naïve glioblastoma
Source: BMC Cancer. 2019 Jun 25;19:628. doi: 10.1186/s12885-019-5861-4 (PMC6593575; doi:10.1186/s12885-019-5861-4)
Supplement: Supplementary file 6 — Validation of selected compounds from the drug screening. We identified drugs with a high DSS and increased patient-specificity (sDSS) and verified the pattern of drug responses in an independent laboratory. (A-C) T1454, (D-F) T1456, and (G-I) T1459. The dose-response curves in the validation experiments are calculated from the mean ± standard error of the mean in five independent experiments and fitted on the basis of a four-parameter sigmoidal logistic fit function. (PDF 342 kb) [file 12885_2019_5861_MOESM6_ESM.pdf]

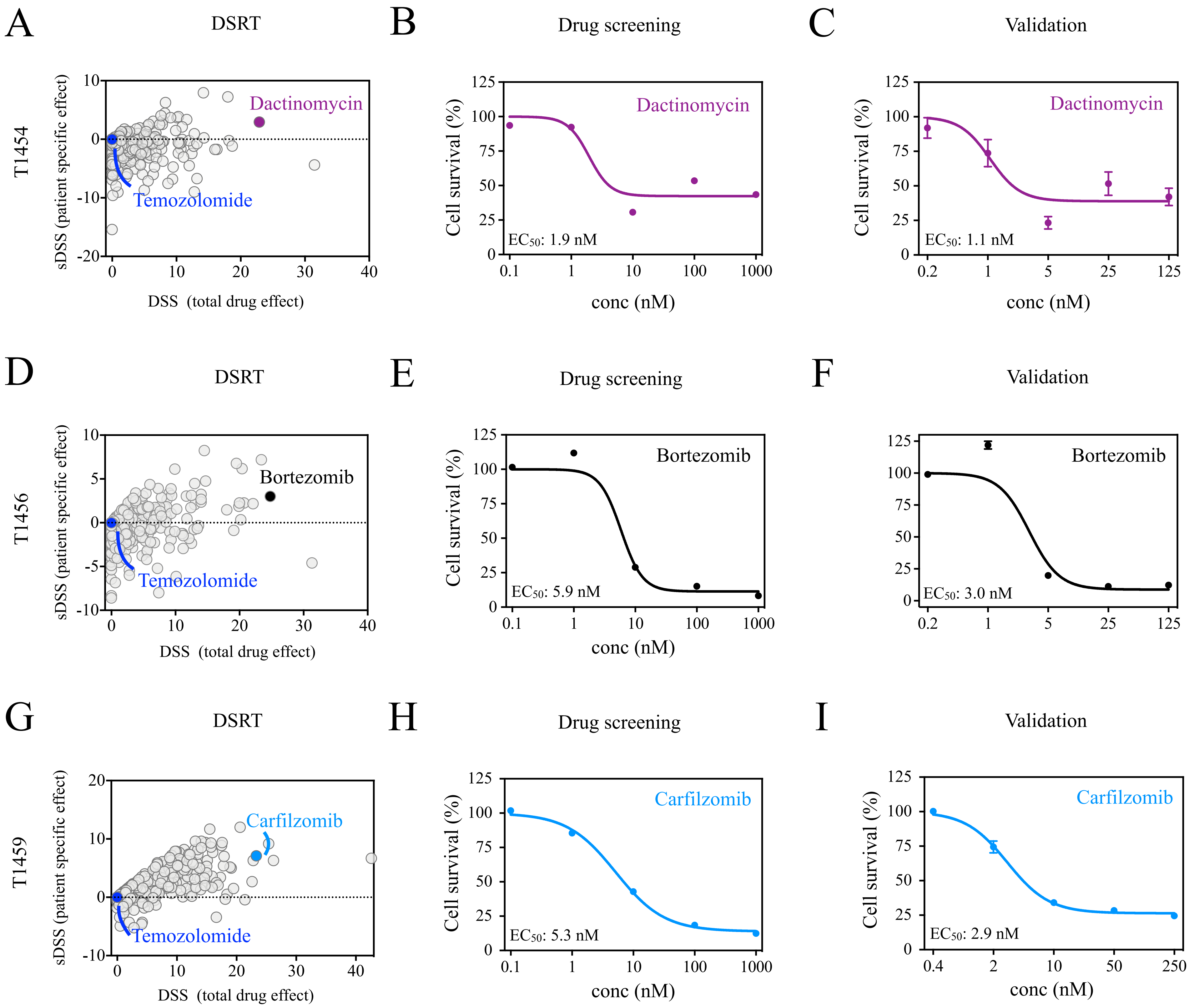
A
DSRT
T1454
sDSS (patient specific effect)
DSS (total drug effect)
Dactinomycin
Temozolomide
B
Drug screening
Cell survival (%)
conc (nM)
Dactinomycin
EC50: 1.9 nM
C
Validation
Cell survival (%)
conc (nM)
Dactinomycin
EC50: 1.1 nM
D
DSRT
T1456
sDSS (patient specific effect)
DSS (total drug effect)
Bortezomib
Temozolomide
E
Drug screening
Cell survival (%)
conc (nM)
Bortezomib
EC50: 5.9 nM
F
Validation
Bortezomib
EC50: 3.0 nM
G
DSRT
T1459
sDSS (patient specific effect)
DSS (total drug effect)
Carfilzomib
Temozolomide
H
Drug screening
Cell survival (%)
conc (nM)
Carfilzomib
EC50: 5.3 nM
I
Validation
Cell survival (%)
conc (nM)
Carfilzomib
EC50: 2.9 nM
