# Supplementary material for: Intertumoral heterogeneity in patient-specific drug sensitivities in treatment-naïve glioblastoma
Source: BMC Cancer. 2019 Jun 25;19:628. doi: 10.1186/s12885-019-5861-4 (PMC6593575; doi:10.1186/s12885-019-5861-4)
Supplement: Supplementary file 9 — Heat map of sDSS in all drugs. Heat map and unsupervised hierarchical clustering of relative effects (sDSS) of the entire drug collection. Gray: failed/missing drug response. (PDF 148 kb) [file 12885_2019_5861_MOESM9_ESM.pdf]

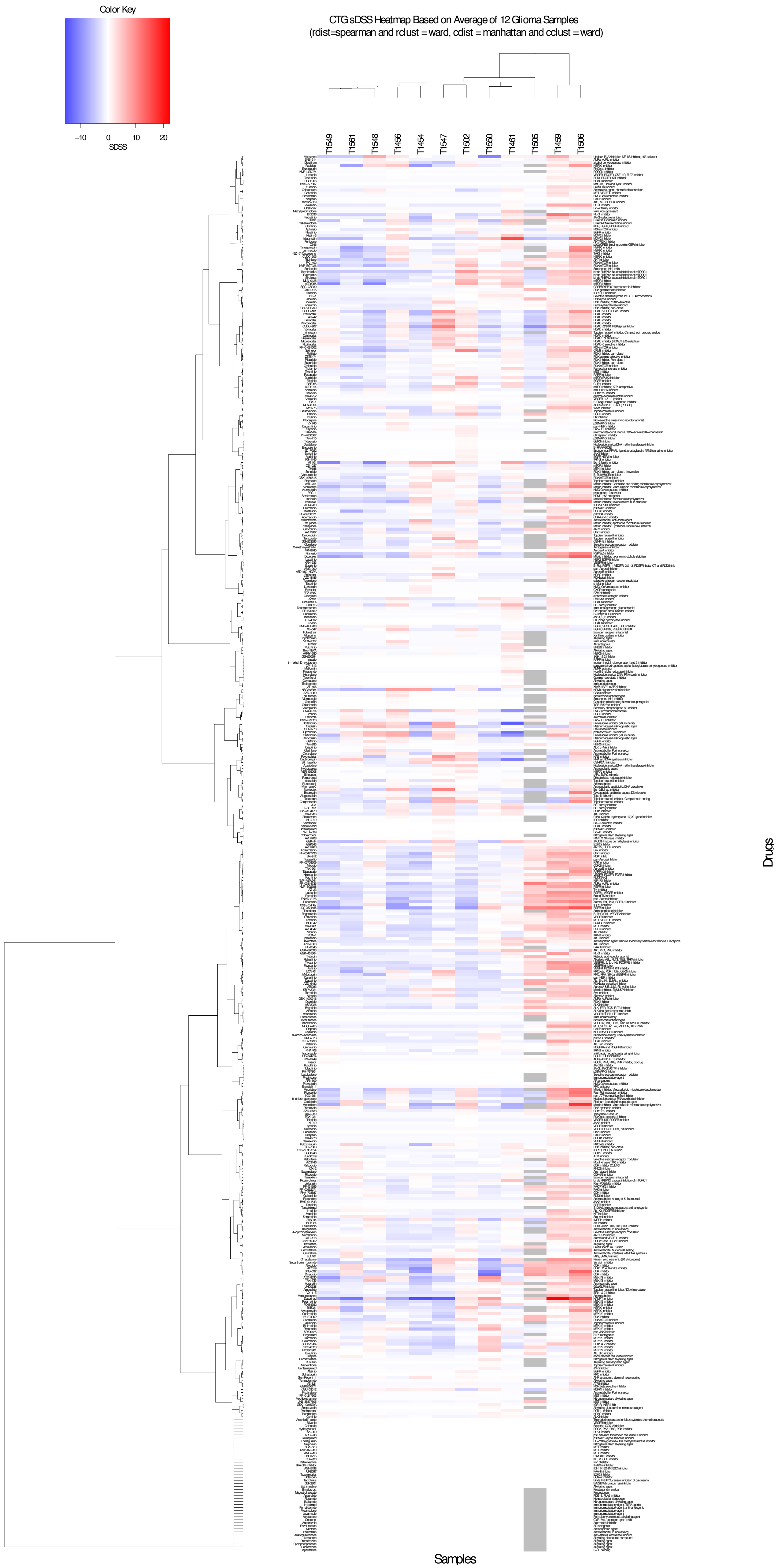

Color Key
−10
0
10
20
SDSS
CTG sDSS Heatmap Based on Average of 12 Glioma Samples
(rdist=spearman and rclust = ward, cdist = manhattan and cclust = ward)
T1549
T1561
T1548
T1456
T1454
T1547
T1502
T1550
T1461
T1505
T1459
T1506
Drugs
Samples
